# Timing and Variability of Galactose Metabolic Gene Activation Depend on the Rate of Environmental Change

**DOI:** 10.1371/journal.pcbi.1004399

**Published:** 2015-07-22

**Authors:** Truong D. Nguyen-Huu, Chinmaya Gupta, Bo Ma, William Ott, Krešimir Josić, Matthew R. Bennett

**Affiliations:** 1 Department of Biosciences, Rice University, Houston, Texas, United States of America; 2 Department of Mathematics, University of Houston, Houston, Texas, United States of America; 3 Department of Biology and Biochemistry, University of Houston, Houston, Texas, United States of America; 4 Institute of Biosciences and Bioengineering, Rice University, Houston, Texas, United States of America; University of Michigan, UNITED STATES

## Abstract

Modulation of gene network activity allows cells to respond to changes in environmental conditions. For example, the galactose utilization network in *Saccharomyces cerevisiae* is activated by the presence of galactose but repressed by glucose. If both sugars are present, the yeast will first metabolize glucose, depleting it from the extracellular environment. Upon depletion of glucose, the genes encoding galactose metabolic proteins will activate. Here, we show that the rate at which glucose levels are depleted determines the timing and variability of galactose gene activation. Paradoxically, we find that Gal1p, an enzyme needed for galactose metabolism, accumulates more quickly if glucose is depleted slowly rather than taken away quickly. Furthermore, the variability of induction times in individual cells depends non-monotonically on the rate of glucose depletion and exhibits a minimum at intermediate depletion rates. Our mathematical modeling suggests that the dynamics of the metabolic transition from glucose to galactose are responsible for the variability in galactose gene activation. These findings demonstrate that environmental dynamics can determine the phenotypic outcome at both the single-cell and population levels.

## Author Summary

Understanding how cells respond to environmental changes is a fundamental question in biology. Such responses are governed by interactions between genes, proteins and other cellular machinery. However, even the responses of genetically identical cells are not identical. Our aim was to examine the origins of this variability using the galactose metabolic network in the baker yeast *Saccharomyces cerevisiae*. This metabolic network allows yeast to consume galactose once its preferred carbon source, glucose, is depleted. We used microfluidic devices and time-lapse fluorescence microscopy to observe how individual cells respond as glucose is removed from their environment at different rates. We found that the activation of the galactose metabolic network depends on the rate of depletion. Surprisingly, cells start to consume galactose faster when glucose is depleted slowly rather than removed quickly. Furthermore, genetically identical cells can exhibit remarkably different rates of galactose consumption. We provide a simple mathematical model that explains these different observations. These results suggest that dynamic changes of environmental conditions can affect the behavior of both individual cells and the whole population.

## Introduction

One way cells respond to environmental changes is by up- or down-regulating genes encoding relevant proteins [1–3]. While the architectures of gene networks (*i.e*. which genes regulate each other under various environmental conditions) have been studied extensively by systems biologists, there is still little known about the dynamics by which cells switch from one gene expression state to another in response to environmental changes. Such dynamics are important, as they can impact cellular fitness and phenotypic diversity [4], and determine downstream pathway activity [5, 6].

In the past, researchers have primarily concentrated on how cells respond to either transient step-wise changes to the environment or periodically varying conditions. For example, it has been shown that the specificity of downstream NF-*κ*B activation depends on the frequency of repeated pulses of tumor necrosis factor-*α* [7]. In yeast, Ronen and Botstein found that multiple dynamic regulatory patterns exist in response to transient pulses of glucose [8]. Other groups have examined how the high-osmolarity glycerol (HOG) pathway in yeast responds to periodic stimuli [9, 10]. Hersen *et al*. found that the HOG pathway acts as a low-pass filter [9], while Mettetal *et al*. showed that various components within the pathway allow the system to respond at different time scales [10]. It has also been shown that the cell cycles of multiple yeast cells can be synchronized by periodically applying pulses of metabolites to the microenvironment [11].

Here, we examine how the galactose utilization pathway in *Saccharomyces cerevisiae* (the GAL network) responds to time-dependent depletion of its repressor, glucose. Regulatory components of the GAL network are responsible for regulating galactose metabolism, and are all up-regulated in the presence of galactose [12, 13]. However, because glucose is the preferred carbon source for *S. cerevisiae*, the presence of glucose tightly down-regulates the GAL network both transcriptionally [14] and post-transcriptionally [15].

The depletion of environmental glucose during yeast fermentative growth triggers a number of changes in cellular activities. The most prominent phenomenon is the inhibition of translation due to the reduction in the number of ribosomes simultaneously translating a singe mRNA [16–18]. In the absence of another carbon source, cells then enter stationary phase and possibly the quiescent state. Specifically, the Snf1p pathway is responsible for activation of catabolism of alternative carbon sources [19]. In the absence of glucose, which leads to an increased AMP:ATP ratio, Snf1p is activated and phosphorylates the transcriptional repressor Mig1p. Importantly for this study, the phosphorylation of Mig1p alleviates its repression of *GAL4*, the master up-regulator of the GAL network, permitting the activation of galactose metabolism [20].

Once glucose has been depleted, and if galactose is available, the activation of the GAL network involves several steps [13]. First, the galactose permease Gal2p imports galactose into cytoplasm. Galactose, along with ATP, activates the galactose sensor Gal3p. Gal3p then binds Gal80p, a transcriptional repressor of the GAL network, and this interaction relieves Gal80p repression on *GAL4* [21–24]. *GAL4* is expressed and drives the expression of several genes in the GAL network, including *GAL2* and *GAL3*. Once the GAL network has been turned on, the first step towards catabolizing galactose is executed by Gal1p, a galactokinase. Hence, activation of *GAL1* is often considered the output of the regulatory pathway [15].

Bennett *et al*. found that, like the HOG pathway, the GAL network acts as a low-pass filter in response to sinusoidal fluctuations of glucose when a constant level of galactose is present [15]. In addition, they were able to use mathematical modeling to correctly predict that certain transcripts within the GAL network were differentially degraded in the presence of glucose. Subsequent experiments showed that GAL transcripts are degraded in the presence of glucose in order to free up resources for cellular division [25]. Interestingly, mutants lacking the ability to differentially degrade GAL transcripts are less fit than wild-type in fluctuating environments, but equally fit in static environments [26].

In the present work we show that the response of the galactose network depends on the rate at which glucose is removed from the environment. Specifically, we used a custom-designed microfluidic device [25, 27, 28] to monitor yeast cells as they were subjected to environments in which glucose was removed at various rates in a background of constant galactose. We found that when glucose is removed gradually from the environment, the GAL network is activated at a fixed time after glucose reached a certain concentration. Furthermore, we observed that the variability of the activation time for the GAL network depends non-monotonically on the glucose depletion rate. While it has been shown that the transition between two metabolic pathways renders cells inactive in a process called diauxie, we show that this phenomenon is most prominent when glucose is depleted quickly. When this occurs, the cell cycle lengths (*i.e*. the time from one division event to the next) can be categorized into three temporally sequential groups: a short cell cycle that occurs while glucose is still present in the growth medium, a highly variable and long intermediate cell cycle that happens during the metabolic transition, and a moderately long cell cycle that occurs once galactose is the sole carbon source. However, when glucose was depleted slowly, we found that the length of the diauxic cell cycle was closer to that of the cell cycle in galactose, which suggests cells smoothly transitioned from glucose to galactose metabolism.

To understand these phenomena, we used computational and statistical models to analyze growth and gene activation of cells in time-dependent environments. We considered different basic models including simple regression, a minimal model based on available energy, and a model incorporating threshold variability in the glucose repression of *GAL1*. Each of these models captures a certain aspect of the activation of the GAL network, but no one model describes the entire process. The results of the modeling suggest that a combination of energy dependence and population heterogeneity were responsible for the non-monotonic variability of the induction times: At very fast glucose depletion times, cells do not have time to make the metabolic transition to galactose before glucose runs out. Since the transition itself requires energy, instantaneous depletion of glucose forces cells into a highly variable, slowly growing state that must be exited for induction of *GAL1*. Conversely, slow glucose depletion allows cells to retain sufficient energy for the metabolic transition and maintain a near-normal growth rate. However, variation in the growth rate among cells leads to a divergence in the level of *GAL1* induction. In general, the transition between repressed and activated stages of the GAL network (or its two bistable stages) is influenced by the depletion rate of glucose.

## Materials and Methods

### Yeast culture and strains

The *Saccharomyces cerevisiae* strain K699-1y (GAL1∷yeVENUS, P_*TEF*1_-HIS5-T_*TEF*1_) was obtained from the Hasty lab [15]. It contains the gene *yeVENUS*, a yeast-enhanced variant of yellow fluorescent protein, fused to the C-terminal of *GAL1* [29]. The strain was grown in YPD (1% yeast extract, 2% peptone, 2% dextrose) or synthetic dropout media minus histidine. For the glucose depletion assays, cells were grown in complete synthetic media (6.7% yeast nitrogen base with ammonium sulfate, standard auxotrophic supplements and appropriate sugar). The inducing medium (hereafter called galactose media) contained 2% galactose (w/v), whereas the repressing medium (glucose media) contained 2% glucose and 2% galactose (w/v).

### Microfluidic device fabrication

The microfluidic device (microfluidic chip, or “chip” for short) used in this study was designed by the Hasty lab [25]. It consists of a cell trapping chamber, five ports, a dial-a-wave (DAW) junction and a staggered herringbone mixer. The height of the trapping chamber is 3.5 *μ*m allowing cells to grow in a single layer. For casting chips, poly-dimethylsiloxane (PDMS, Sylgard 184, Dow Corning) was mixed in 1:10 ratio (catalyst:base), degassed and cast against a master mold. The solution was cured at 80°C for 1 hr in a dry oven. The five ports were created by punching the PDMS monolith using a 0.5 mm puncher (Harris Uni-Core, Sigma-Aldrich). Chips were cleaned by sonication in methanol. Using UV/ozone treatment, a chip and a glass coverslip were exposed to UV light and ozone in a UVO cleaner (Jelight Company Inc.) for 3 min. Treated surfaces were brought into contact within 1 min and allowed to anneal at 80°C overnight to enhance bond strength [28]. They were stored at room temperature until subsequent experiments.

### Glucose depletion assays

One day prior to each glucose depletion assay, a single colony was inoculated in 5 mL of galactose media and grown at 30°C at 250 rpm overnight. On the day of each experiment, 1 mL of overnight culture was transferred into 50 mL of glucose medium and grown to OD_600_ 0.3–0.6 (∼ 8 hr at 30°C, 250 rpm) before loading into the microfluidic chip. The setup of the microfluidic device and the time-lapse fluorescence microscopy have been described previously [15, 28]. Briefly: A microfluidic chip was first primed with water. Five reservoirs were connected to the chip using 0.02in x 0.06in Tygon Micro-Bore tubing (Saint Gobain Performance Plastics) with 23-gauge Luer stub adapters (Becton Dickinson) and connection pins. Two reservoirs contained 20 mL of galactose and glucose media. Fluorescent tracer dye (Sulforhodamine 101, 1 *μ*g/ml, Invitrogen) was added to glucose media to track the environmental concentration of glucose [15]. Two other reservoirs acted as waste outlets. The fifth reservoir contained the yeast culture. The chip was left in the growth chamber, attached to the microscope, for 30 min to allow it to reach thermal equilibrium at 30°C. Cells were loaded into the cell trapping chamber and were imaged every 5 min for 15 h in red, yellow fluorescence and transmitted light while a preprogrammed controller dynamically changed the input media (see below).

### Depletion of environmental glucose

The GAL network was induced through a steady depletion of environmental glucose. For this purpose, reservoirs containing the inducing and repressing media were fixed onto two linear actuators (ROBO Cylinder, Intelligent Actuator Inc.) [15, 28]. Prior to loading cells onto the microfluidic device, the objective lens was focused on the upstream DAW junction on the device. The presence of fluorescent dye was measured (Sulforhodamine 101, 586nm/605nm) and its signal at the DAW junction was used to calibrate the positions of the two actuators through a custom Labview program named iDAW (National Instruments, developed in the Hasty lab at UCSD) [15, 28]. Once the positions were calibrated, a set of commands was supplied to iDAW to control the ratio of environmental glucose and galactose inside the chip. This set included 3 steps; first, maintaining glucose at 2% for 4 hr; second, linearly reducing the glucose concentration from 2% to 0% at various rates; and third, maintaining glucose concentration at 0% for the remainder of the assay. At each step in the process the galactose concentration was held constant at 2%. In one control we maintained glucose for 6 hr (instead of 4 hr) before beginning the depletion of glucose. In another, no glucose was ever present.

### Data analysis

For each microscope experiment, time-lapse images of each fluorescent channel were loaded into ImageJ. The mean of red fluorescence was measured to verify the environmental concentration of glucose. Segmentation of single cells was performed using phase contrast images and a contrast enhancement algorithm (Watershed). We obtained changes in fluorescent intensity of Gal1p-YFP in each cell by using phase contrast images to track single cells throughout the image series. Using phase contrast image sequences, budding events for each cell were recorded when a bud emerged from the mother cell. Consequently, the “cell cycle length” was defined to be the time between two successive budding events.

### Mathematical simulations

We used the delayed Stochastic Simulation Algorithm (dSSA) for simulating the dynamics of the GAL network [30]. The dSSA was modified by limiting the maximum allowed time increment to 1 minute. This allowed us to use reaction propensity functions that vary with time (in order to correctly incorporate a time-varying metabolic rate for the cells due to changing energy availability) [31]. The delay was assumed to only influence reactions that resulted in the production of protein. In simulations we used a fixed delay of 5 minutes.

Cell volume for each cell was initialized to *V*(0) = 1, and was explicitly modeled by introducing a term *V*(*t*) = exp(*γ*_*t*_ *t*). The rate of cell growth, *γ*_*t*_, itself varied in time due to differences in energy availability. Once the cell volume reached 2, a cell was assumed to divide and the volume was reset to 1. Proteins, both mature and immature, were assigned to the daughter cell randomly according to a binomial distribution with parameter *p* = 0.5.

To perform our data analysis and model fitting, we needed to infer, from experimental data and simulated fluorescence trajectories, the carbon source being utilized by the cell. We used a heuristic rule for this purpose. We retained only those cells that completed six division events. Any cell in which the last observed cell division event corresponded to the longest observed cell cycle was considered to not have exited the diauxic lag phase, and was ignored in subsequent analysis. The cell cycles for the remaining cells were progressively clustered into three non-overlapping groups. The groups were initialized by choosing the mean *μ*_*Glu*_ to be the length of the first observed cell cycle length, *μ*_*Gal*_ to be the length of the last observed cell cycle length and *μ*_*Diauxie*_ to be the largest observed cell cycle length. If the previous cell cycle was determined to belong to the Glucose group, the next cell cycle was classified as Glucose or Diauxie depending on which group it was closer to. However, if the previous cycle was classified as Diauxie, the next cycle was assigned to Diauxie or Galactose. This heuristic rule was used to analyze both simulated and experimental data. We observed that in the experimental data, the largest cell cycle usually occurred right around glucose depletion.

## Results

**Fig 1:**
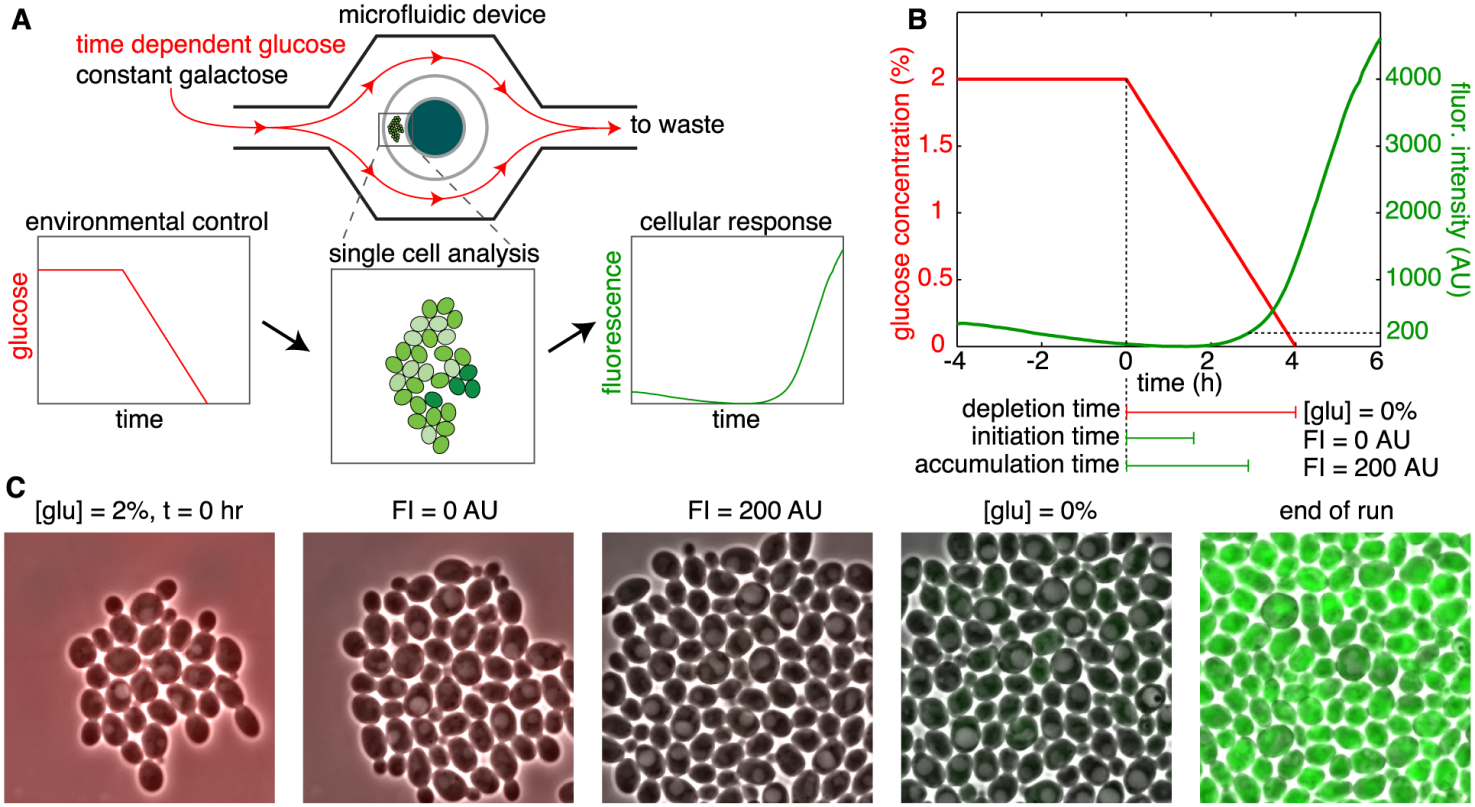
Experimentally tracking single cells during glucose depletion assays. A) Cells were trapped within a microfluidic flow chamber and the environmental concentration of glucose was depleted as a function of time, while holding the galactose concentration constant. As glucose levels dropped, individual cells heterogeneously activated Gal1p production, and the resulting fluorescence trajectories were recorded. B) Glucose concentration as a function of time (red line). Here, the depletion time is 4 hrs. Also shown is the experimentally measured fluorescence trajectory of an individual cell to the 4 hr depletion time (green line). This cell first initiates Gal1p production and then accumulates protein (below). C) Images of yeast cells in the microfluidic device at successive events from a 4 hr glucose-depletion assay. [glu] = 2%, t = 0 (hr) indicates the beginning of glucose depletion; [glu] = 0% indicates total depletion of glucose; labels FI = 0 and 200 (AU) correspond to the times at which FI reaches these values; end of run, the end of glucose depletion assay.

To investigate the effect of glucose depletion rates on the induction of the galactose network, we used *S. cerevisiae* strain, K699-1y, that contains a *YFP* fusion to the *GAL1* gene [15]. Uninduced cells were loaded into a previously described microfluidic device that permits continuous culture of a monolayer of yeast and precise temporal control over the growth medium [25]. During loading and set-up of the microfluidic device, cells were exposed to media containing 2% galactose and 2% glucose. Fig 1 depicts the general procedure of the experiments. At the beginning of each trial, we began to decrease the concentration of glucose at a constant rate until it reached zero, while maintaining a constant level of galactose. Due to this change, cells eventually switched from glucose metabolism to galactose metabolism. To determine the times at which cells turned on the galactose network, we monitored the YFP fluorescence of individual cells which served as a proxy for *GAL1* activity (Fig 1A).

In each experiment, there are three times that are of particular interest (Fig 1B). First is the “depletion time”—the time between the beginning of glucose depletion until its concentration reaches zero. Second, the “initiation time” is the time at which fluorescence in a cell first begins to increase. At the beginning of the experiment, each cell has higher fluorescence intensity (FI) than the background. This is expected since the cells have a history of galactose metabolism. We observed that, during glucose depletion, FI values slowly decrease before increasing again as cells acclimated to the galactose environment. We call the time at which a FI trajectory changes from having a negative to a positive slope the “initiation point.” We rescaled the FI so that it equals zero at the initiation point. We called the interval between the beginning of glucose depletion and the initiation point the “initiation time” (Fig 1B). Finally, the “accumulation time” is the interval between the initiation of glucose depletion and the time at which cell fluorescence reaches an arbitrary threshold above that at the initiation point (Fig 1B).

**Fig 2:**
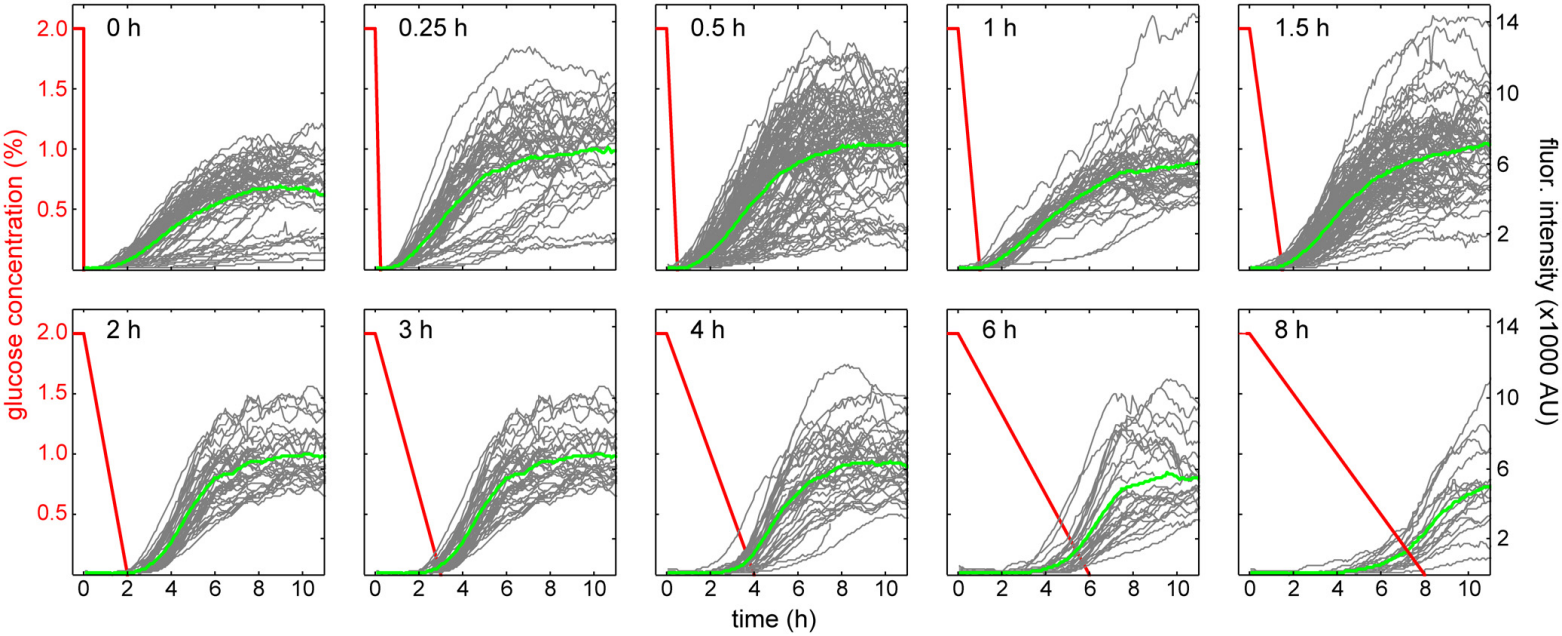
Experimentally measured single-cell responses to different glucose-depletion times. Ten glucose-depletion times were tested. Red lines depict changes in glucose concentration from 2% to 0% (w/v). Gray curves depict individual fluorescence trajectories of Gal1-YFP. Green curves show mean FI. For reference, the fluorescence of cells grown in just galactose was approximately 8200 ± 2000 AU (S1 Dataset, sheet 14).

We tested ten different depletion rates, varying the depletion time between 0 and 8 hrs. We chose this range of depletion times to cover both unnatural induction events, in which glucose is quickly removed from a culture to induce protein expression, and more naturally occurring scenarios, in which glucose is more gradually depleted by a growing colony. Even when glucose is being naturally depleted from the environment, it has been shown that cells exhibit heterogeneity in their growth rates under diauxic shift [32, 33]. Fig 2 shows representative single-cell YFP fluorescence trajectories of YFP-tagged Gal1p and time-dependent concentrations of environmental glucose for each of the depletion times tested (S1 Dataset, sheet 1–11).

**Fig 3:**
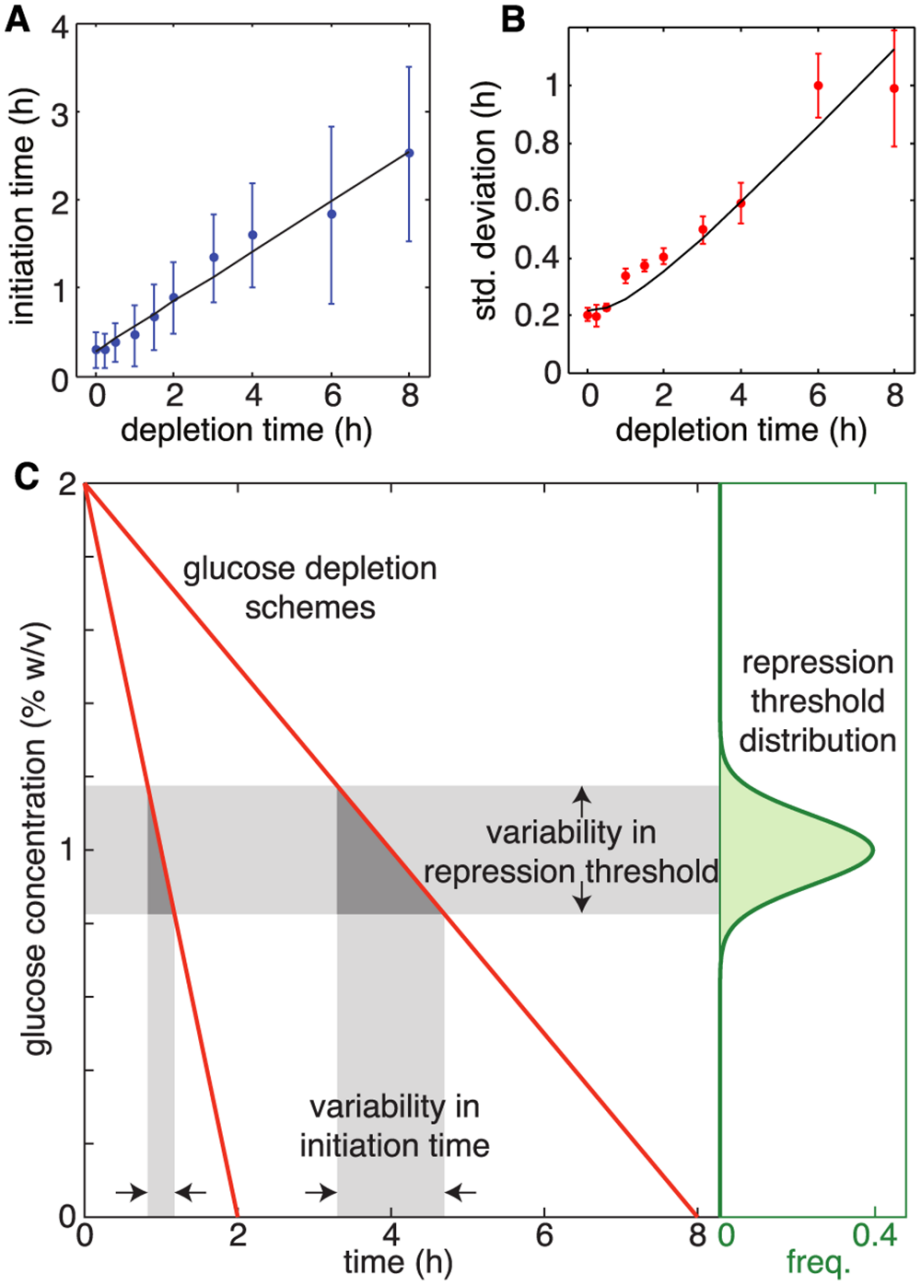
The initiation of *GAL1* expression depends linearly on the glucose depletion time. A) The mean initiation time increases linearly with increasing depletion time. Shown are the mean (dots), standard deviations (error bars), and best fit line. B) The standard deviation of the initiation time is related to the variability of the threshold g* and the delay time *τ* according to Eq (2). Error bars represent the standard deviation of the measurement as calculated with a bootstrapping method. C) The dependence of both the mean and the standard deviation of the initiation time can be explained by a simple threshold model. As glucose depletes (red curves, left) a variable threshold (green distribution, right) is crossed that relieves repression of the galactose genes. The slower the transition through the repression thresholds (horizontal shaded region) the greater the variability in the initiation time (vertical shaded region).

When we plotted the mean and standard deviation of the initiation times against the depletion times, we observed that the initiation time was linearly related to the depletion time (Fig 3A, R^2^ = 0.974). This result fits an intuitive hypothesis about GAL network activity: The GAL network activates some time, *τ*, after glucose has dropped below a threshold concentration, g*. This simple model would indicate that the initiation time obeys

$$T_{i}=[\frac{g_{s}-g^{*}}{g_{s}}]\;T_{d}+\tau ,$$ (1)

where *T*_*i*_ is the initiation time, *T*_*d*_ is the depletion time, and *g*_*s*_ is the initial glucose concentration (here, *g*_*s*_ = 2%). Fitting Eq 1 to data gives us approximate values for the threshold and the delay time, namely g* ≈ 1.4% (*P* < 0.001, 95% CI = (1.17%, 1.63%) and *τ* ≈ 17 min. (*P* < 10^−7^, 95% CI = (15.4 min, 18.6 min)). Furthermore, the standard deviation of the initiation time as a function of depletion time (Fig 3B) suggests that the threshold concentration of glucose and the delay time vary from cell to cell. As depicted in Fig 3C, the time it takes to sweep past the distribution of thresholds increases as the depletion time increases. Therefore, assuming that variations in *τ* and g* are independent, this simple model predicts that the standard deviation of the initiation times is given by

$$\sigma^{2}\left(T_{i}\right)=\frac{\sigma^{2}\left(g^{*}\right)}{g_{s}^{2}}T_{d}^{2}+\sigma^{2}\left(\tau \right),$$ (2)

where *σ*(g*) is the standard deviation of the repression threshold, and *σ* ^2^(*τ*) is the variability in the activation delay. Indeed, our data support this relationship between $T_{d}^{2}$ and *σ* ^2^(*T*_*i*_), and fitting Eq 2 to the data gives us *σ*(g*) ≈ 0.2% and *σ*(*τ*) ≈ 18 min.

Note that the initiation time for the 0 hr depletion was not significantly different when we held cells at 2% galactose for 6 hr (instead of 4 hr) before the depletion of glucose. With a 6 hr “pre-growth,” the initiation time was 0.35 ± 0.21 hr, compared to an initiation time of 0.30 ± 0.20 hr with a 4 hr pre-growth (S1 Dataset, sheet 12–13).

We next examined how the accumulation time depends on the depletion time. While the initiation of genes within the GAL network is an important step in the process of changing from glycolysis to galactose metabolism, the proteins encoded by the GAL genes must accumulate before full galactose metabolism can occur. To measure this, we first chose an arbitrary threshold FI value that was above the background level, but still well below the mean steady-state levels. The interval between the initiation point and the time at which a cell’s FI trajectory first reached this threshold value was recorded as the accumulation time.

**Fig 4:**
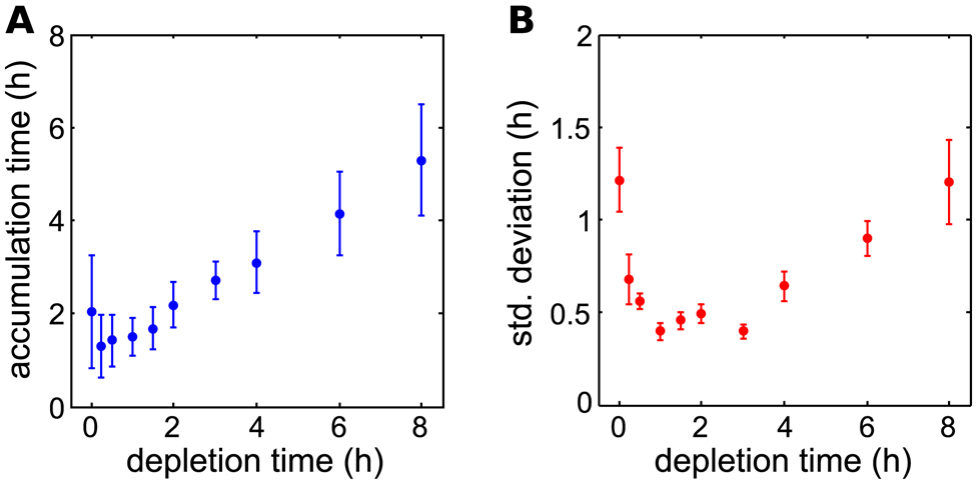
Gal1p accumulation changes non-monotonically with glucose depletion rate. A) Average accumulation time as a function of the depletion time (error bars represent SD). B) The standard deviation of the accumulation time as a function of the depletion time. In fast and slow depletion schemes, the accumulation of Gal1p is highly variable, and achieves a minimum at intermediate depletion rates. Error bars represent the standard deviation of the measurement as calculated with a bootstrapping method.

As a function of the depletion time, the accumulation time looks similar to the initiation time (Fig 4). However, there are two important differences. First, the accumulation time is larger for near instantaneous than for intermediate depletion times. This indicates that cells accumulate proteins more quickly if glucose is depleted slowly rather than instantaneously. Second, the standard deviation of the accumulation time is not an increasing function of the depletion time.

We next wanted to understand why the accumulation time behaved differently than the initiation time when glucose was depleted rapidly. One piece of evidence was cell cycle length. We noticed that cells exposed to rapid glucose depletion temporarily stopped growing after glucose was totally depleted. By contrast, cells experiencing more gradual depletion did not exhibit this behavior. We hypothesized that the cessation of growth during fast depletion times represented a diauxic shift as the cells transition from glucose metabolism to galactose metabolism. For yeast cells growing on a single carbon source, diauxie is a hallmark of the stationary and quiescence phase, when cells exhaust their preferred carbon source and start to metabolize another. As a result, cells delay or stop their division to conserve energy while their translational machinery is poised to exit diauxie when new carbon sources become available. We further hypothesized that during these slow cell cycles, protein production within the GAL network became very slow. Therefore, even though the network might be initiated, the cells did not have sufficient energy to produce GAL network proteins rapidly, leading to accumulation times that were much longer than normal.

**Fig 5:**
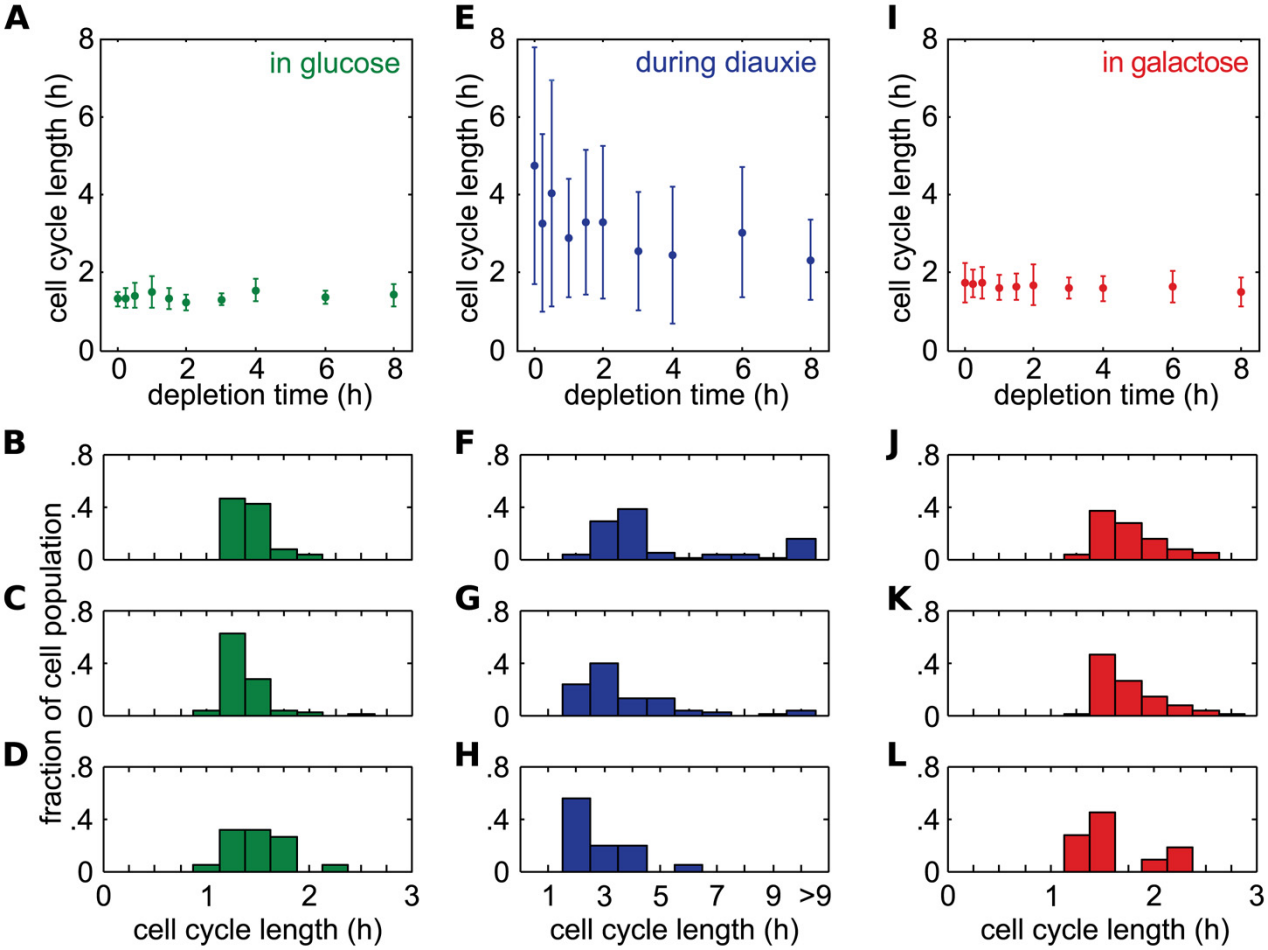
Cell cycle times during glucose depletion. A) Mean and standard deviation (error bars) of cell cycle times during growth in glucose (before glucose depletion) for different depletion times. B- D) Histograms of the cell cycle times shown in (A) for three different depletion times (0 hr, 2 hr and 8 hr, respectively). E-H) Same as (A-D), but during growth in the diauxic transition between glucose and galactose (during glucose depletion). G-L) Same as (A-D), but for growth in galactose (after glucose depletion).

To better characterize how the glucose depletion rate influences the growth dynamics of cells during the diauxic shift, we tracked the budding events of individual cells throughout each experiment, and calculated the cell cycle lengths (the time from one budding event to the next) of each cell (Fig 5). We observed that, before glucose depletion, the average cell cycle length was 1.38 ± 0.25 hr (Fig 5A). Well after glucose was completely depleted and the cells were growing only in galactose, the average cell cycle length was 1.65 ± 0.38 hr (Fig 5I). These two times represent normal growth in glucose and galactose media, respectively. However, between these two extremes, cell cycle lengths tended to be longer and highly variable, especially for fast depletion times (Fig 5E).

The longest cell cycles occured just as glucose was nearly depleted and we used these cell cycle lengths as estimates of the diauxic cell cycle lengths. We found that the length of the cell cycle during the diauxic phase was, on average, 3.19 ± 1.95 hr. However, the diauxic cell cycle length depended greatly on the depletion time. For instance, the diauxic cell cycle length was longest for instantaneous depletion times, at 4.74 ± 3.04 hr, and shortest for a depletion time of 8 hrs, at 2.32 ± 1.03 hr. Additionally, the SD of the diauxic cell cycle length was largest for instantaneous depletion times, at 3.04 hr (Fig 5B). Thus, while galactose is always present in the environment, the transition from glucose to galactose metabolism can be dictated by the depletion rate of glucose. Specifically, instantaneous depletion of glucose appeared to have two correlated effects: 1) an increase in the accumulation time, and 2) an increase in the diauxic cell cycle length.

It should be noted that those cells that exhibited the largest cell cycle times (> 9 hr, *e.g*. Fig 5F) during diauxie also had large accumulation times. In fact, when these cells were removed from the 0 hr depletion time data, the average accumulation time of the remaining cells is significantly shorter (1.78 ± 0.92 hr), and better fits into the overall linear relationship between accumulation time and depletion time (R^2^ = 0.97).

The simple model given in Eq (1) cannot explain why the cell cycle lengths vary with the rate of glucose depletion, nor can it explain the non-monotonicity in the accumulation times or their SD with increasing depletion times (Fig 4B). We therefore next wanted to use a simple mathematical model that could explain: 1) the depletion time dependence of the diauxic cell cycle length, 2) the increase in accumulation times for short depletion times, and 3) the non-monotonicity of the SD of the accumulation times as a function of depletion time.

### An energy model for GAL network induction

When glucose depletion occurs, the repression on the GAL network is released; however, achieving full induction requires translation of multiple regulatory components of the network. Since translation is known to require up to 50% of the cell’s free energy [34], incorporating energy availability into our model may help us understand the behavior of the network. Further, a lack of available energy is what causes the diauxic lag [35]. We therefore hypothesized that the noisy intermediate cell cycle lengths, as well as the initial decrease in the variability of the accumulation times of the GAL network with increasing glucose depletion times, are due to the more gradual changes in energy availability to the cell which occur at longer depletion times.

**Fig 6:**
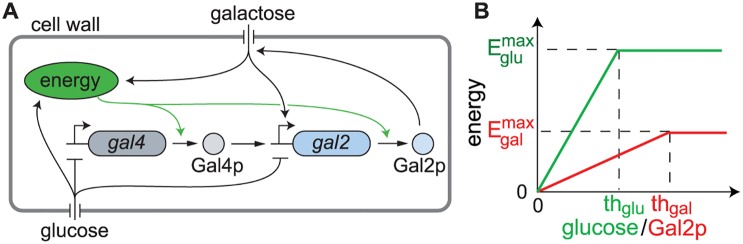
An energy model for the glucose/galactose switch. A) A schematic of the regulation in the heuristic stochastic model. Glucose inhibits expression of the GAL genes, but increases the cellular energy needed for protein production. Gal4p up-regulates transcription of *gal2*, whose gene product, Gal2p, imports galactose. The ability of the cell to metabolize galactose is therefore dependent on the availability of Gal2p. In addition, intercellular galactose increases cellular energy and up-regulates *gal2*. B) We used piecewise linear functions for *E*_*glu*_([*glu*]) and *E*_*gal*_([*Gal*2*p*]). The *E*_*glu*_ term is assumed to only depend on the environmental glucose concentration. The *E*_*gal*_ term does not depend on environmental galactose because it is held constant; what changes is the amount of galactose that is utilized by the cell, a good proxy for which is the concentration of Gal2p. The maximum (steady-state) energy levels $E_{glu}^{max}$ and $E_{gal}^{max}$ are inferred from the cell-cycle lengths in the two conditions. The threshold *th*_*glu*_ is the glucose threshold at which the cell is assumed to obtain maximal energy. The equivalent threshold for galactose is *th*_*gal*_; this is the threshold at which the cell has sufficient Gal2p to maximally utilize environmental galactose.

To test our hypothesis, we derived a minimal model of the GAL network including only two proteins, Gal2p and Gal4p, as well as a unitless time scaling parameter, *E*, that depends on the energy available to the cell. This energy term reflects the basal metabolic rate of the cell under different carbon sources (Fig 6). We used the time-delayed stochastic simulation to simulate realizations of the model given by the reactions

$$\emptyset \overset{E\beta_{4}}{\to }g_{4}$$ (3)

and

$$\emptyset \overset{E\beta_{2}}{\to }g_{2},$$ (4)

where

$$\begin{array}{ccc} \beta_{4} & = & \frac{\alpha_{4}}{1+{\left(glu/c_{4}\right)}^{n_{4}}} \end{array}$$ (5a)

$$\begin{array}{ccc} \beta_{2} & = & \frac{\alpha_{2}{\left(g_{4}/c_{2}\right)}^{n_{2}}}{1+{\left(g_{4}/c_{2}\right)}^{n_{2}}} \end{array}$$ (5b)

$$\begin{array}{ccc} E & = & \max\{E_{glu}\left(\left[glu\left(t\right)\right]\right),E_{gal}\left(g_{2}\right),\epsilon \}. \end{array}$$ (5c)

Here, *g*_4_ and *g*_2_ are the concentrations of Gal4p and Gal2p, respectively, and *α*_4_ and *α*_2_ are their respective maximal production rates; Gal4p production is repressed by glucose, with a Hill coefficient *n*_4_ and a half-maximal concentration *c*_4_; Gal2p production is activated by Gal4p, with a Hill coefficient *n*_2_ and half-maximal concentration *c*_2_; *E*_*glu*_([*glu*(*t*)]) is the energy time scaling due to glucose metabolism, and depends on the time-dependent concentration of glucose, [*glu*(*t*)]; *E*_*gal*_(*g*_2_) is the energy time scaling due to galactose metabolism, which is a function of the concentration of Gal2. Both *E*_*glu*_([*glu*(*t*)]) and *E*_*gal*_(*g*_2_) are piecewise-linear functions of their arguments, increasing from zero until reaching a maximum (representing a maximum metabolic flux rate which depends on the carbon source, see Fig 6). Note that Gal2p is the transporter for galactose, and hence intracellular galactose concentrations will depend on *g*_2_. In this manner, Gal2p provides positive feedback for the galactose network. In addition, when energy from both glucose and galactose is low, we assume that the time scaling parameter is at a minimum, *E* = *ϵ*. This represents a basal metabolic activity of the cells after glucose has been depleted but before there is enough Gal2p to effectively transport galactose into the cells.

The time varying rate, *γ*_*t*_, at which cells grow was computed as *Eγ* where *γ* is a basal growth rate. Since the energy term *E* scales the cell growth rate, the maximum levels $E_{glu}^{max}$ and $E_{gal}^{max}$ were fit to the experimentally measured cell cycle lengths in glucose and galactose, respectively. In addition, since *ϵ* represents the growth rate in the diauxic phase (which was highly variable), we sampled *ϵ* from a Beta distribution on the interval (0, *ϵ*_max_) with two shape parameters and the range parameter *ϵ*_max_. We obtained the range and shape parameters for this distribution by fitting the model to the cell cycle lengths observed experimentally.

The constant *c*_4_ in Eq (5a) is the glucose concentration at which the GAL network is half-maximally repressed. While functionally different from the g* term in Eq (1), any variability in the threshold at which glucose repression on the GAL network is released will affect both *c*_4_ and g* in the same way. In order to make the stochastic model consistent with the simple model from Eq (1), we sample *c*_4_ from a normal distribution. The contribution of the variability in *c*_4_ is analogous to that of g* in Eq (1); variability in the accumulation times increases with increasing depletion times.

**Fig 7:**
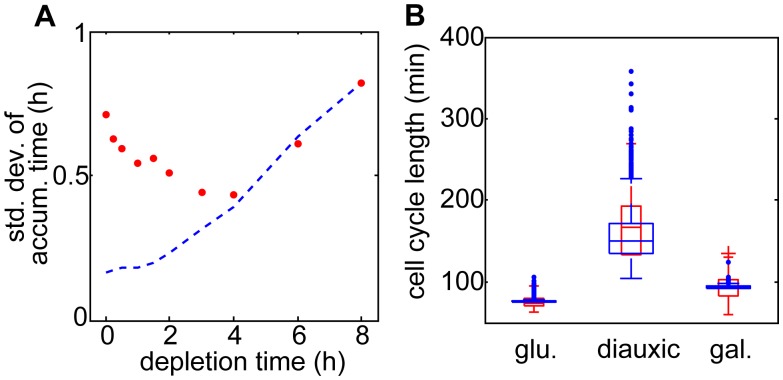
Results of the mathematical model. A) Simulated standard deviation of the accumulation time as a function of the depletion time for the full model (red dots) and a model lacking the energy scaling (blue dashed line). Note that the energy scaling recreates the non-monotonicity observed in the experiments. B) Box plots of the distributions of the different types of cell cycle lengths for the 3 hr depletion scheme. The red box plots are the experimental data, whereas the blue box plots are obtained from a simulation of Eq. (5). The distribution of the energy availability in the diauxic phase (*ϵ*) is obtained by fitting the model to the diauxic cell cycle data.

Our mathematical modeling suggests the non-monotonicity in the variance of accumulation times is a consequence of the variability in the energy available to cells when they transition between carbon sources. To test whether this was indeed the driving force behind the non-monotonicity in the model, we also fit a model in which the scaling term *E* is set to a constant value independent of the carbon source being utilized. Our model shows that in the absence of a time-varying energy term *E*, the standard deviation of the accumulation time monotonically increases with the depletion time (blue dashed line, Fig 7A). When the scaling term *E* is allowed to vary depending on the glucose concentration and Gal2p accumulation, the non-monotonic behavior observed experimentally is recreated (red dots, Fig 7A).

## Discussion

It is often thought that the presence of glucose causes tight repression of the GAL network and that this repression is only alleviated when glucose is nearly depleted from extracellular environment. Recent investigations on wild and laboratory yeast strains, however, showed that the GAL network can be activated much earlier and in the presence of glucose [32, 36]. Furthermore, Escalante-Chong and colleagues showed that the induction of the GAL network depends on the ratio of glucose and galactose and is independent of glucose depletion [37]. Based on this ratio, at galactose concentration of 2% (w/v), a glucose concentration less than 1% (w/v) can trigger the onset of the GAL network’s induction within 1 hr [37]. Consistent with their results, our data showed that the GAL network is initiated approximately 15 min after glucose concentration reaches 1.4% (w/v), in the context of a 2% galactose background (Fig 3). While the mean of the initiation time is independent of the depletion rate, we found that its variability does depend on the depletion rate, which dictates the time cells move through this glucose concentration threshold. Interestingly, the increase in the variability of the initiation time is consistent with Young *et al*., in which they showed that the rate of change of an environmental signal can affect the variability in the expression of *σ* ^*B*^ factor in *E. coli* [38].

The onset of the GAL network’s induction, determined by the initiation time, is not strongly affected by glucose depletion rate. However, we found that the accumulation of Gal1p significantly depends on glucose depletion rates. We showed that the accumulation time of Gal1p is larger for near instantaneous depletion times than for intermediate depletion times (Fig 4A). Furthermore, the standard deviations of the accumulation times follow a non-monotonic relationship with the depletion times (Fig 4B). These results, coupled with a long, highly variable diauxic length of yeast cells in near instantaneous depletion times, suggest a detrimental effect on the accumulation of the GAL network proteins due to a significant decrease in intracellular energy resource (Fig 5B).

Several energy factors could contribute to the delay in Gal1p accumulation for fast glucose depletion times. First, it has been shown that yeast cells regulate intracellular activities based on the perceived concentration of extracellular glucose [39]. Thus, instantaneous depletion of glucose could lead to a large energy waste on ribosomal biogenesis (which is needed for exponential growth in glucose) and thus lead to growth arrest in yeast cells [39]. Second, Venturelli *et al*. recently showed that, while the GAL network can activate in the presence of glucose, cells do not significantly consume and metabolize galactose until glucose is depleted [36]. Consequently, cells lack energy to sustain exponential growth. As a result, the combined affect of these two factors could result in significant delay and high variability in Gal1p accumulation times. The most severe consequence is the growth-arrested quiescence state of a subset of the population. This is confirmed by our data and was previously demonstrated in both yeast and bacteria [33, 40, 41].

To deal with environmental uncertainty, microbes have evolved various mechanisms to regulate cellular activities to adapt to environmental changes, including bet-hedging and stochastic sensing [33, 40, 41]. The high variability of Gal1p accumulation times in response to the instantaneous depletion of glucose seen here might be evidence of stochastic expression of the GAL network. The result is two distinct subpopulations of cells: growth-arrested cells that can quickly return to high growth rates if glucose reappears and fast growing cells that have a high growth rate in the new environment. If so, changes in the variability of Gal1p accumulation times might reflect the cost-benefit tradeoff employed by yeast cells in fluctuating environments [32, 36], and the ability to stochastically switch between the two phenotypes might provide a fitness advantage [42].

Overall, our data showed that the rates of glucose depletion can significantly affect the expression of the GAL network. While the GAL network can be initiated very quickly and reliably independent from glucose depletion, it is the intracellular energy resource, influenced by glucose depletion rates, that dictate the accumulation and full induction of the GAL network within each individual cell of the population. These findings demonstrate that environmental dynamics can determine the phenotypic outcome at both the single-cell and population levels.

## Supporting Information

S1 Dataset Complete dataset. Spreadsheet containing data from each microscope run described in this article.
(XLSX)
